# Acting on the CFTR Membrane-Spanning Domains Interface Rescues Some Misfolded Mutants

**DOI:** 10.3390/ijms232416225

**Published:** 2022-12-19

**Authors:** Nesrine Baatallah, Ahmad Elbahnsi, Benoit Chevalier, Solène Castanier, Jean-Paul Mornon, Iwona Pranke, Aleksander Edelman, Isabelle Sermet-Gaudelus, Isabelle Callebaut, Alexandre Hinzpeter

**Affiliations:** 1 INSERM, U1151, Institut Necker Enfants Malades (INEM), Faculté de Médecine, Université Paris Cité, CNRS, UMR 8253, 75015 Paris, France; 2 Sorbonne Université, Muséum National d’Histoire Naturelle, UMR CNRS 7590, Institut de Minéralogie, de Physique des Matériaux et de Cosmochimie, IMPMC, 75005 Paris, France

**Keywords:** CFTR, ABC transporter, ABCC, ABCB, structure function relationships, molecular dynamics, cystic fibrosis, pharmaco-chaperone

## Abstract

ABC transporters are large membrane proteins sharing a complex architecture, which comprises two nucleotide-binding domains (NBDs) and two membrane-spanning domains (MSDs). These domains are susceptible to mutations affecting their folding and assembly. In the CFTR (ABCC7) protein, a groove has been highlighted in the MSD1 at the level of the membrane inner leaflet, containing both multiple mutations affecting folding and a binding site for pharmaco-chaperones that stabilize this region. This groove is also present in ABCB proteins, however it is covered by a short elbow helix, while in ABCC proteins it remains unprotected, due to a lower position of the elbow helix in the presence of the ABCC-specific lasso motif. Here, we identified a MSD1 second-site mutation located in the vicinity of the CFTR MSD1 groove that partially rescued the folding defect of cystic fibrosis causing mutations located within MSD1, while having no effect on the most frequent mutation, F508del, located within NBD1. A model of the mutated protein 3D structure suggests additional interaction between MSD1 and MSD2, strengthening the assembly at the level of the MSD intracellular loops. Altogether, these results provide insightful information in understanding key features of the folding and function of the CFTR protein in particular, and more generally, of type IV ABC transporters.

## 1. Introduction

The ABC transporters share a common architecture made of two nucleotide-binding domains (NBDs) and two membrane-spanning domains (MSDs), the latter having distinct folds depending on the ABC subtype considered [1]. The biogenesis of these large membrane proteins is a complex, multistep process which involves insertion of transmembrane (TM) helices into the lipid bilayer, folding of individual domains and inter-domain assembly. Mutations can alter these steps, leading to defective expression, activity and/or localization. Disease-causing variants that affect the folding have been identified in different ABC transporters. This is the case, for instance, with ABCB4 [2] and ABCB11 [3], which are associated with cholestatic liver diseases. This is also the case for the cystic fibrosis transmembrane conductance regulator (CFTR or ABCC7), in which mutations cause cystic fibrosis (CF), a severe genetic disease affecting around 70,000 patients world-wide. The fundamental knowledge of the folding mechanisms is probably the most extensive for CFTR, with detailed investigations of the folding steps occurring co- and post-translationally [4,5,6,7,8,9,10,11,12,13,14].

**Figure 1:**
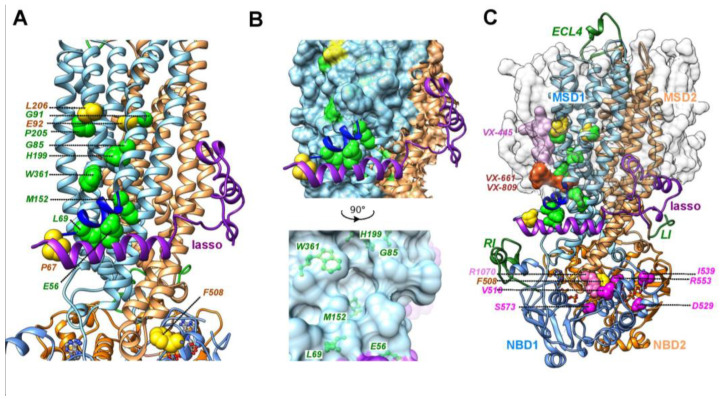
Position of MSD1 class II mutations, F508del, and F508del-revertants. (**A**) Focus on the positions of MSD1 amino acids for which class II mutations were observed, shown on the experimental 3D structure of human CFTR (pdb:7SV7, ribbon representation). The position of F508 is shown as a reference. All these amino acids are shown in green, except for P67, E92, L206 and F508 (in yellow), which were analyzed in this study. ATP molecules are shown in a ball-and-stick representation. MSD1/NBD1 are shown in light blue, with the MSD1 elbow helix depicted in dark blue. The lasso is show in purple. MSD2/NBD2 are shown in light orange. (**B**) Focus on the groove displayed at the membrane inner leaflet (surface representation), in which are found several class II mutations (in green), and which can be occupied by pharmaco-chaperones VX-661/VX-809. (**C**) Positions of the amino acids for which mutations allow the rescue of the F508del defect (F508del revertants), located in NBD1 and ICL4 (R1070). The protein was embedded in a phospholipid (POPC) bilayer (grey surface), with models of the RI (Regulatory Insertion), the LI (Linker Insertion) and the ECL4 (ExtraCellular Loop 4) shown in dark green. The binding site of VX-661/VX-809 in MSD1 is shown in orange, according to [22,23], while that proposed for VX-445 [22] is highlighted in light pink.

Mutations affecting CFTR folding and maturation (referred to as class II mutations) have been mapped in the different domains of CFTR, namely within the N-terminal lasso motif, the MSDs (illustrated for MSD1 in Figure 1A), the NBDs, and the intracellular loops (ICLs), linking the MSDs to the NBDs. These mutations include the most frequent cystic-fibrosis-causing mutation F508del located within the NBD1 domain, which was shown to affect both NBD1 thermostability and NBD1-ICL4 assembly [9,11,12,13,14].

Interestingly, the F508del maturation defect can be partially rescued by second-site mutations (Figure 1C). These mutations, named revertants, affect several amino acids located in NBD1: V510D [15], I539T [16], G550E [16], R553M/Q [17], and D529F or S573E [9], as well as a basic amino acid located in ICL4 at the interface with NBD1 (R1070W) [13]. Mutations initially introduced to enhance recombinant NBD1 solubility and facilitate production and purification steps also rescued cell surface expression of the full-length CFTR-F508del protein (F494N/Q637R and F429S/F494N/Q637R [18]). Partial rescue was also obtained by deleting specific regions and motifs, such as the regulatory insertion (RI, F405–L436) from NBD1 [19] or RXR ER-retention motifs (R29K, R516K, R555K, and R766K, termed 4RK [20]). Revertants have been suggested to act by different mechanisms to repair structural defects caused by the F508del mutation, leading to enhanced ER exit and improved NBD1 stability or inter-domain contacts between NBD1-NBD2 or NBD1-ICL4 [21].

No such revertant has been identified to date for class II mutations located within the N-terminal lasso or MSD1 domain, which present a wild-type NBD1 and ICL4 sequence, such as the CF-causing P67L, E92K, and L206W (Figure 1A). Folding of the CFTR MSD1 constitutes a limiting step in the CFTR biogenesis [7]. The correctors Lumacaftor/Tezacaftor (VX-809/VX-661) developed to rescue misfolded CFTR-F508del directly bind within a groove of MSD1 at the level of the inner membrane leaflet (Figure 1B,C) [22,23], improving the overall stability of this domain, and in turn, rescuing most of the mutations located in MSD1. These class II mutations are located within or in the vicinity of this groove. Another binding site was also highlighted in MSD1 for Elaxacaftor (VX-445) at the level of the membrane outer leaflet, connected to the Lumacaftor/Tezacaftor binding site and allowing the two corrector types to act in an additive way [22]. By an allosteric spillover effect, binding of the corrector to MSD1 also favors inter-domain assembly and correction of F508del defects. Of note, F508del correction also required corrector binding onto NBD1, which is thermodynamically unstable, in contrast to MSD1 mutants [22]. Here, we report on MSD1 L188W, which was identified as a revertant of the folding defect of mutant L206W but not of mutant F508del, when exploring possible binding sites of pharmaco-chaperones [22]. Identifying such revertant(s) of class II mutations specific of MSD1 may therefore reveal important features of the CFTR dysfunction and MSD1-specific mechanisms involved in correction.

## 2. Results

### 2.1. L188W Rescues MSD1 Class II Mutation L206W

**Figure 2:**
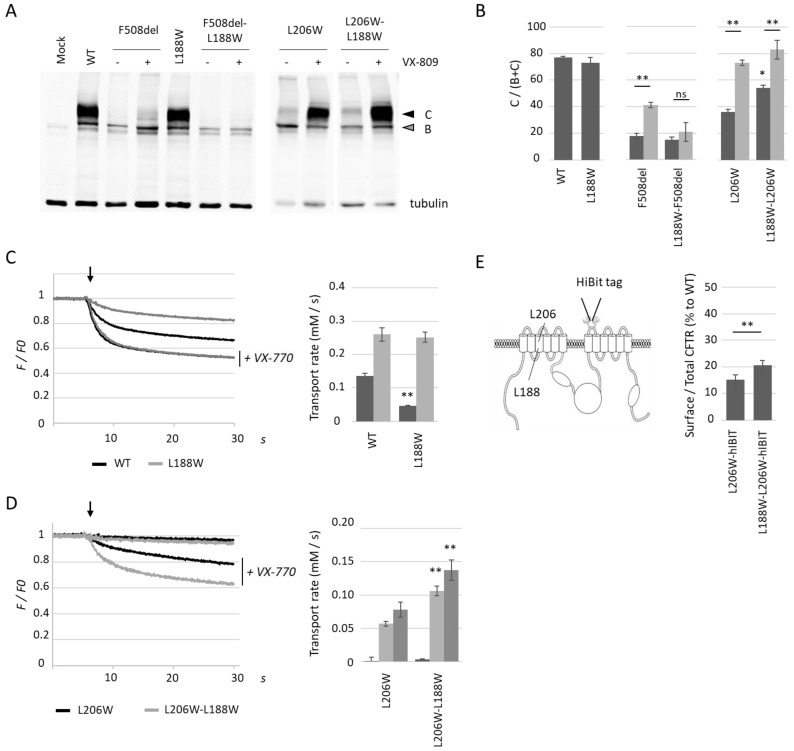
L188W partially reverts the folding defects of L206W but not F508del. (**A**) Western blot analysis of proteins obtained from HEK293 cells transiently transfected with the indicated CFTR mutant with and without VX-809 (3 µM, 24 h). Arrows indicate mature fully glycosylated CFTR (dark, band C) and partially glycosylated CFTR (grey, band B). Tubulin was used to assess equal loading. (**B**) Quantification of the C/(B + C) maturation ratio from the western blot analysis of the indicated mutants. Dark grey represents control conditions and light grey VX-809 treatments. Measures are means ± SEM of *n* = 5–10, with * indicating *p* < 0.05, ** indicating *p* < 0.01. (**C**,**D**) Representative recordings using the halide sensitive YFP assay obtained from HEK293 cells expressing indicated CFTR mutant before and after addition of iodide-containing PBS (injection indicated with an arrow), with and without the CFTR potentiator VX-770 (10 µM). Histograms represent mean ± SEM of calculated transport rates for the indicated mutant. In (**C**), dark grey represents cAMP cocktail induced activation (*n* = 5) and light grey cAMP + VX-770 (*n* = 5) and in (**D**) dark grey represents cAMP cocktail induced activation (*n* = 7), light grey cAMP + VX-770 (*n* = 6) and medium grey cAMP + VX-770 + apigenin (*n* = 3) with for both, * indicating *p* < 0.05, ** indicating *p* < 0.01 and ns non-significant. (**E**) Cell surface expression of L206W and L206W-L188W normalized to the total CFTR in the cell and compared to CFTR-WT (*n* = 5) with ** indicating *p* < 0.01. Measures were performed using the hIBIT complementation assay with the hIBIT tag inserted between amino acids Ser898 and R899.

Within the L206W background, we identified L188W as a MSD1 located revertant. Analysis by western blot using proteins obtained from HEK293 cells transiently transfected with plasmids encoding either wild-type (WT) or mutant L188W CFTR indicated that single-mutation L188W did not alter protein maturation as assessed by the ratio of mature to total CFTR protein (C/(B + C) maturation ratio) (Figure 2A,B). The activity of the CFTR-L188W was measured using the halide sensitive YFP functional assay [24] after co-transfection of plasmids encoding WT or mutant CFTR and yellow fluorescent protein variant YFP-H148Q/I152L. In this assay, CFTR was first activated by PKA phosphorylation via cAMP-elevating cocktail, and then iodide ions are added. The entry of iodide ions via CFTR induces the quenching of the YFP-H148Q/I152L fluorescent probe, reflecting channel activity. Compared to CFTR-WT, CFTR-L188W was found to be less active upon application of cAMP elevating cocktail (cpt-AMPc and IBMX, 100 µM each). Addition of potentiator VX-770 (10 µM) fully rescued CFTR-L188W activity, which reached CFTR-WT levels in the same conditions, reminiscent of a gating defect.

L188W was then introduced in both CFTR L206W and F508del plasmids. In the F508del context, L188W did not affect maturation and even prevented VX-809 correction (Figure 2A,B). Results were markedly different in the L206W context, with L206W-L188W showing a significantly higher basal maturation ratio as compared to L206W, while both L206W and L206W-L188W were fully corrected with VX-809 (Figure 2A,B). CFTR-L206W-L188W showed a higher activity compared to CFTR-L206W upon application of cAMP-elevating cocktail and potentiator VX-770 (10 µM) (Figure 2D), consistent with increased numbers of channels at the cell surface. This could also be observed in the presence of co-potentiator apigenin (20 µM), which further activated CFTR channels (Figure 2D). To quantify CFTR cell surface expression, a hIBIT tag sequence composed of amino acids VSGWRLFKKIS was inserted in the 4th extracellular loop of CFTR (ECL4). The association between the hIBIT tag and the luciferase large subunit enabled the reconstitution of an active enzyme and quantification of CFTR cell surface amounts. Subsequent permeabilization of cell membranes using Triton X-100 enabled the entry of the luciferase large subunit into the cell and the quantification of total CFTR. Results obtained using this procedure showed greater surface expression for double mutant L206W-L188W (21 ± 1.4% of WT, *n* = 5) as compared to single mutant L206W (15 ± 1.9% of WT, *n* = 5) (Figure 2E). Taken together, these results identify L188W as a folding revertant specific to L206W located in the MSD1 domain of CFTR.

### 2.2. L188W Acts on the MSD1:MSD2 Interface

**Figure 3:**
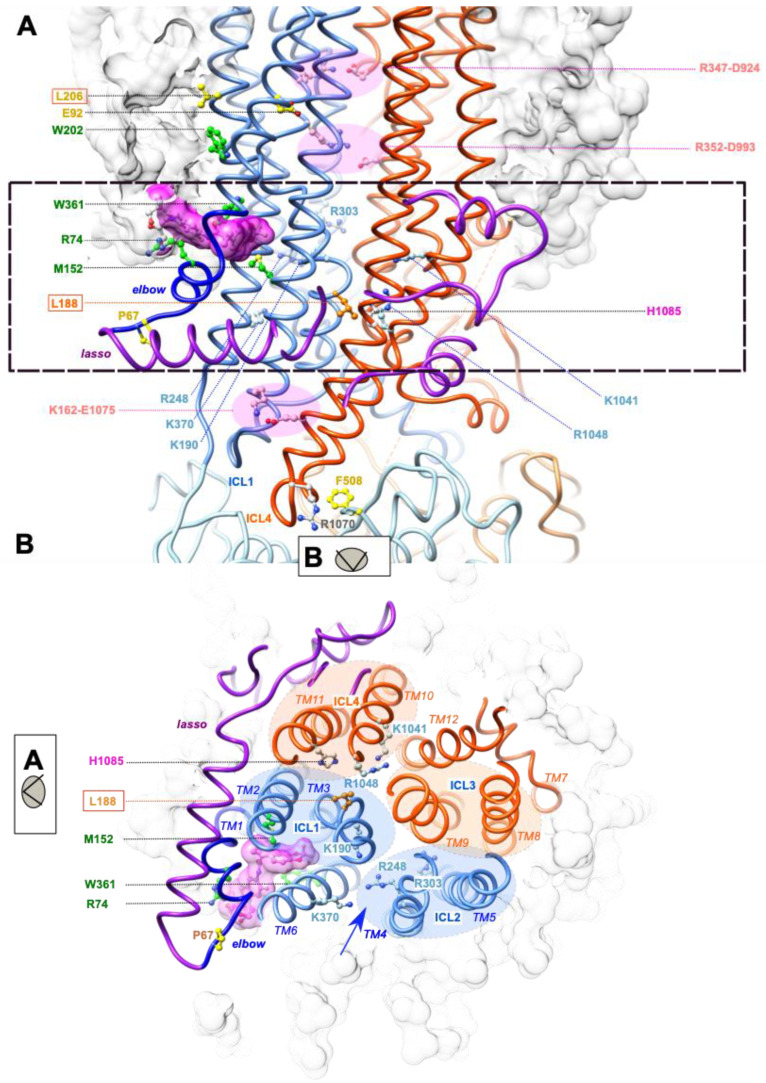
The L206W and L206W-L188W mutations on the 3D structure of human CFTR. (**A**,**B**) A 3D structure of CFTR-WT in complex with VX-661 (pdb:7SV7), taken as a reference and embedded in a model lipid bilayer (POPC, grey surface). The two views are orthogonal. The slice shown in panel (**B**), viewed from the NBDs, is highlighted with a dashed bow in panel (**A**). Depicted in atomic detail, amino acids of the VX-661 binding pocket (green), affected by class II mutations (yellow), basic amino acids of the cytoplasmic portal and vestibule [28,29] (blue), and amino acids involved in salt-bridges [22,26] (pink).

**Figure 4:**
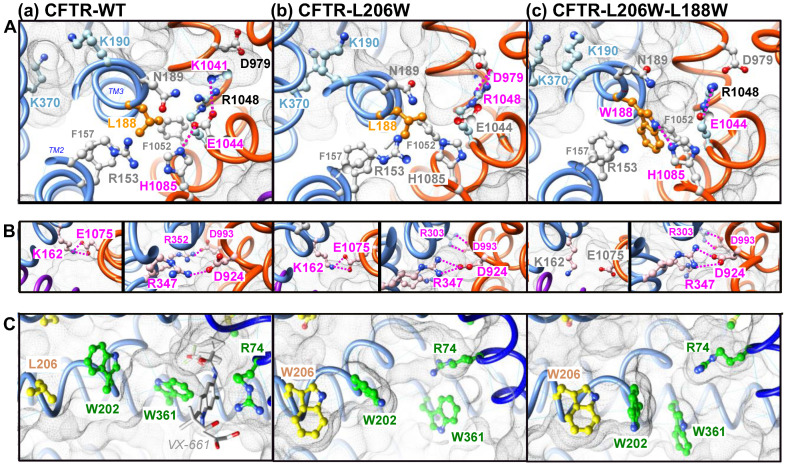
(**A**–**C**) Comparison of the 3D structures of CFTR-WT (**a**), CFTR-L206W (**b**), or CFTR-L206W-L188W (**c**), with focuses on the L188 neighborhood (**A**), the salt-bridges (**B**), and the relationship between L206W and the binding groove (**C**). The 3D structures of the mutated proteins are illustrated here after 700 ns MD simulations (starting from the 3D structure of human CFTR (pdb:6MSM)). Amino acids linked by H-bonds or salt bridges are depicted in pink.

Our previous study provided insights into how the L206W mutation, located in transmembrane helix TM3 above the Lumacaftor/Tezacaftor binding pocket (Figure 3A), may affect the overall stability of MSD1 [22]. Indeed, a short (125 ns) molecular dynamics (MD) simulation indicated that L206W places the W202 side chain away from the position it occupies in the wild-type protein, which is at the edge of the binding pocket and in contact with W361. As a result, the W361 side chain is moved out of the pocket, leaving room for lipids that are stabilized by multiple contacts, but probably leading to an overall lower stability of the domain. L188 is located far away from L206, within the cytoplasmic extension of transmembrane helix TM3 (Figure 3A), at a level where ICL1 (formed by the cytoplasmic continuations of MSD1 TM2 and TM3) and ICL4 (formed by the cytoplasmic continuations of MSD2 TM10 and TM11) tightly interact (Figure 3B). The side chain of L188 is oriented opposite the Lumacaftor/Tezacaftor binding pocket and is located under the main lateral portal, allowing ion flow from the cytoplasm (between TM4 and TM6, including K370) and under the floor of the pore vestibule (R303) (Figure 3A,B). L188 is buried within a hydrophobic environment in the vicinity of F157 (MSD1 TM2) and F1052 (MSD2 TM10) (Figure 4C(a)).

We performed here long (700 ns) MD simulations of the CFTR-WT, CFTR-L206W and CFTR-L206W-L188W proteins in order to understand how L188W might increase the overall stability of the mutated protein. Figure 4 shows the 3D structures at the end of the simulations, highlighting specific features that are mostly stably observed throughout the simulation. The mutation of leucine in tryptophane allowed a new H-bond to be made between its Nε1 atom and the Nε2 atom of H1085, located in MSD2 TM11 (Figure 4A(c)). This novel and stable interdomain interaction, with the external helix of ICL4, likely increased the overall stability of MSD1. This novel bond was accompanied by a modification of the interaction network. Indeed, H1085 was interacting in the CFTR-WT with E1044, itself forming a salt bridge with K1041 (Figure 4A(a)). In CFTR-L206W-L188W, this network was lost, and E1044 formed another salt bridge with R1048 (Figure 4A(c)) when R1048 was not bound to D979, as was the case with CFTR-L206W (Figure 4A(b)). Further analysis of the MSD1-MSD2 interface in CFTR-L206W-L188W revealed the frequent disruption of the K162 (ICL1 TM2)-E1075 (ICL4 TM11) salt bridge, which allosterically connected MSD1 with NBD1 [22] (Figure 4B—left views). Of note (Figure 4B—right views) is that only one (R347-D924) of the two salt bridges (R352-D993 and R347-D924) required for normal channel function [25,26,27] was maintained in the two mutated proteins, while an alternative bond was formed between D993 (in salt bridge with R352 in CFTR-WT) and R303, which left its central position in the vestibule of the WT protein.

### 2.3. L188W Rescues Other Class II Mutations Localized within the Lasso Motif or MSD1

We further explored the effect of L188W on class II mutants P67L in the N-terminus of CFTR and E92K located in TM1 (Figure 1A). A similar mechanism of action as for L206W could be expected for the P67L mutation, located at the external edge of the Lumacaftor/Tezacaftor binding pocket. In contrast, the E92K mutation appeared per se more difficult to rescue, as this amino acid is involved in a salt bridge with K95 in the same TM1 helix, which stabilizes it and allows proper insertion into the membrane.

**Figure 5:**
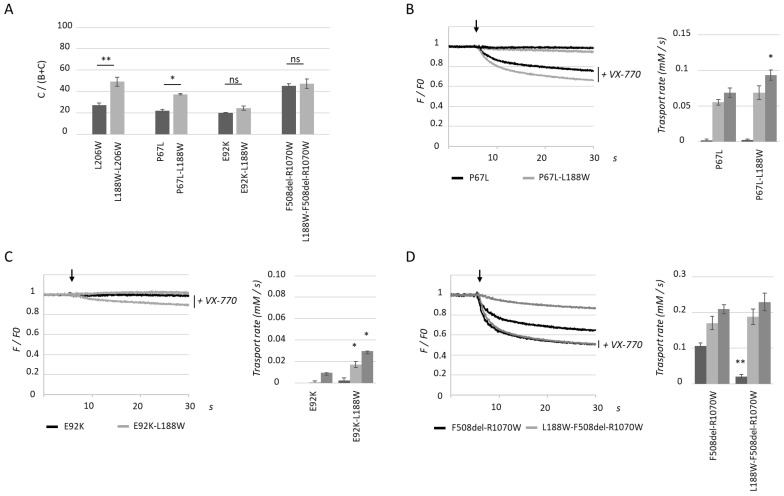
L188W rescues other MSD1 mutants but not F508del-R1070W. (**A**). Quantification of the (**C**)/(**B**,**C**) maturation ratio based on immunoblot analysis of proteins obtained from HEK293 cells transiently transfected with the indicated single (dark grey) and double (light grey) mutants. Measures are means ± SEM of *n* = 5–10, with * indicating *p* < 0.05 and ** indicating *p* < 0.01. (**B**–**D**). Representative recordings using the halide sensitive YFP assay obtained from HEK293 cells expressing indicated CFTR mutants before and after addition of iodide-containing PBS (injection indicated with an arrow), with and without the CFTR potentiator VX-770 (10 µM). Histograms represent mean ± SEM of calculated transport rates for the indicated mutant (*n* = 3–4), with * indicating *p* < 0.05 and ** indicating *p* < 0.01. Dark grey represents cAMP activation, light grey cAMP + VX-770 (10 µM) and medium grey cAMP + VX-770 (10 µM) + Apigenin (20 µM).

Quantification of western blot analysis of proteins obtained from transiently transfected HEK293 cells showed that P67L and E92K maturation was altered as expected, with low C/(B + C) ratios as compared to WT CFTR. As observed with L206W, P67L maturation was also increased by secondary mutation L188W, but to a lesser extent (Figure 5A). Consistent with this result, CFTR-P67L-L188W showed statistically significantly increased activity upon application of cAMP and the VX-770 with apigenin combination, but not with cAMP and VX-770 alone (Figure 5B). For CFTR-E92K-L188W, the application of VX-770 potentiated a low activity which was further enhanced by apigenin, even though the surface expression ratio was not significantly enhanced by the second site mutation (Figure 5C). Finally, L188W was tested on double mutant F508del-R1070W, where R1070W partially corrects NBD1-ICL4 assembly. As with single mutant F508del, L188W did not enhance maturation of CFTR-F508del-R1070W (Figure 5A). Using the halide sensitive YFP assay, the activity of CFTR-L188W-F508del-R1070W was significantly lower than CFTR-F508del-R1070W (Figure 5D). Upon application of VX-770 or the VX-770/apigenin combination, transport rates were not different between the two mutants, consistent with results obtained in the WT context.

Taken together, these results indicate that L188W acts as a revertant specific to some class II mutants located in the N-terminus of CFTR.

### 2.4. The MSD1 Instability Is Likely Associated with the Presence of an ABCC-Specific Lasso Motif

**Figure 6:**
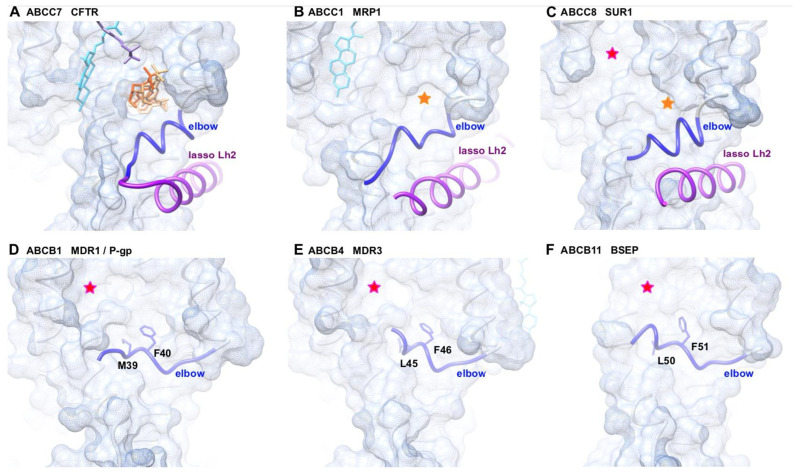
Comparison of the MSD1 from type IV exporters of the ABCC and ABCB families. Surface representation, after superimposition, of the experimental 3D structures of three members of the ABCC subfamily: ((**A**) human CFTR, pdb 7S7V; (**B**) bovine MRP1, pdb 6VY0; (**C**) human SUR1, pdb 7S5X) and three members of the ABCB subfamily; ((**D**) human ABCB4, pdb 6S7P; (**E**) human ABCB11, pdb 7DV5; (**F**) human ABCB1, pdb 6C0V). The lasso motifs and elbow helices, which have not been considered for surface calculation, are colored in purple and blue, respectively. The conserved aliphatic–aromatic motif of ABCB proteins is shown in atomic detail, together with the position of VX-661 (Tezacaftor) in the CFTR 3D structure (red and orange wire for the experimental 3D structure (pdb 7S7V) [23] and docking/MD simulations/site directed mutagenesis [22], respectively). Orange stars highlight the position of the corresponding groove in other ABCC proteins, while the pink stars pinpoint another groove present in type IV ABC protein, which is occupied in some of them by cholesterol (light blue). The position of VX-445, as proposed in [22] is depicted in purple on the CFTR 3D structure.

The groove of MSD1, in the vicinity of which are located several class II mutations (Figure 1A,B), is occupied by the correctors Lumacaftor (VX-809) – Tezacaftor (VX-661) (Figure 1C) [22,23]. It can thus be considered as a fragile region of the protein, concentrating mutations and stabilized by the action of pharmaco-chaperones. It is lined by TM1, TM2, TM3, TM6 and the elbow helix (in dark blue on Figure 1), which runs parallel to the TM helices and is located at the interface between the phospholipid bilayer and the cytosol. This elbow helix is in tight contact with a cytosolic alpha-helix (Lh2) belonging to an ABCC-specific motif called “lasso” (in purple on Figure 1). In particular, a salt-bridge is observed in CFTR between the Lh2 E56 and the elbow R75 [22]. Similar grooves are observed for members of the ABCC subfamilies, for which 3D structures have also been solved, such as human MRP1 (ABCC1) [30] and human SUR1 (ABCC8) [31] (Figure 6).

We then compared this region with type IV ABC proteins lacking the lasso, such as members of the ABCB subfamily. Several 3D structures are available for this subfamily, including ABCB1 (MDR1 or P-glycoprotein) [32], ABCB4 (MDR3) [33], and ABCB11 (ABCBB, BSEP) [34], for which several patient-derived variants have been described (MIM:61224, MIM:602347, and MIM:601847, respectively). Examination of these 3D structures (Figure 6) indicated that grooves similar to those observed in ABCC exporters were indeed also present in these ABCB exporters, these being more or less wide and/or deep. However, in these ABCB examples, the elbow helix covered the lower part of the groove, while in the ABCC examples, the elbow helix appeared pulled down to the cytosol, interacting with the lasso helix Lh2. Interestingly, this lower position of the elbow helix enabled accessibility to the pharmaco-chaperone VX-809 binding site, while this site was occupied by a conserved phenylalanine in ABCB examples, provided by the elbow helix. The fact that VX-809 and the phenylalanine occupied a similar position suggests that protection provided by the elbow helix in these ABCB proteins could stabilize MSD1, contrasting with the unprotected status of the ABCC proteins.

## 3. Discussion

The CFTR MSD1 domain harbors a groove which concentrates multiple class II mutations, suggesting that it constitutes a fragile region of the protein. This groove accommodates pharmaco-chaperones [22,23], which stabilize the global MSD1 fold. We show that MSD1 contains a second site mutation (or revertant), L188W, which was able to partially rescue L206W and to a lesser extent P67L and E92K, likely favoring the assembly and stability of MSD1. Revertants have been widely considered to increase the fundamental knowledge of the processing, trafficking, stability, and function of the CFTR-F508del and other CF mutants (e.g., [13,15,16,17,18,19,21,35,36,37]). However, until now and to the best of our knowledge, none has yet been mapped in MSD1. Such revertants thus provide a valuable tool for studying the characteristics of this region, whose folding and stability is critical for the whole architecture of the protein. Interestingly, the L206W revertant L188W had no effect on CFTR-F508del, suggesting that the beneficial effect could not be transmitted to other domains of the protein. In the same logic, we also tested the effect of F508del revertants R555K (NBD1) and R1070W (ICL4) in the L206W context and found that while R1070W had no effect, R555K favored the appearance of band C. This latter result could be associated with enhanced ER exit induced by the disruption of the RXR retention motif in position 553–555, regardless of the CF mutation, while R1070W did not enhance ICL4-NBD1 association when NBD1 was not mutated.

According to the results obtained by 3D structure modeling, L188W participated in the MSD1-MSD2 interface at the level of the ICLs, enabling some contacts and disabling others. This could be observed at the level of the L188W itself, which formed a novel H-bond with H1085. This novel bond probably reinforced the MSD1-MSD2 assembly leading to the stabilization of mutated MSD1, hence the revertant effect of L188W. While L188W induced a novel H-bond with H1085, it also disrupted a salt bridge connecting K162 (ICL1) and E1075 (ICL4), as compared to the wild-type situation and the L206W single mutation. This salt-bridge was previously shown to be critical for the rescue of NBD1-located mutant F508del via an allosteric coupling between the MSD1-located VX-809 binding site and NBD1 [22]. Disruption of this salt bridge explains the lack of rescue of CFTR-L188W-F508del by the pharmaco-chaperone VX-809 or of CFTR-F508del-R1070W, where R1070W partially rescued the ICL4-NBD1 assembly. Other bonds at the MSD1:MSD2 interface were also found to be disrupted, such as a salt bridge between R352 and D993. These bonds are considered to be good indicators of the channel open state [26], and their disruption could explain the reduced activity measured for CFTR-L188W as compared to WT channels. This is supported by the same amounts of mature CFTR (band C) and equal cell surface expression measured between WT and L188W CFTR. As F508del-R1070W and L188W-F508del-R1070W also showed equal amounts of band C, it is likely that alterations in the H-bond network also occurred in the F508del-R1070W background. On another hand, one cannot exclude an effect on the anion entry in the pore, as L188 is in the vicinity of basic amino acids located in the cytoplasmic lateral portal and in the pore vestibule (e.g., K190, R248, R303, K370). This reduced activity was however fully corrected with the CFTR potentiator VX-770.

The comparison of members of the ABCC and ABCB families highlights the complex influence that the C-terminal part of the lasso (Lh2) may have on the first membrane-spanning domain, by “pulling down” the elbow helix relative to the position it adopts in ABCC proteins and thereby, leaving “unprotected” curved areas formed by the assembly of transmembrane helices. At the same time, the lasso appeared to be a key actor for the assembly with ICL1 [7], and by a knock-on effect, with ICL4 and NBD1.

This study highlights the usefulness of comparing structures from different members of the ABC superfamily. Experimental 3D structures are becoming increasingly available, moreover in different conformational states, thanks to developments in cryo-electron microscopy techniques. Their analysis, combined with molecular dynamics simulations in relevant membrane bilayer models, could reveal key regions implicated in their folding or activity.

## 4. Materials and Methods

### 4.1. Plasmids and Mutagenesis

The cDNA of CFTR WT (M470) was subcloned in pTracer as previously published [38]. Mutagenesis of all the indicated mutants was performed using the QuickChange XL II mutagenesis kit (#200522-5, Agilent, Les Ulis, France) following the manufacturer’s instructions. The hIBIT tag was inserted between amino acids S898 and R899. Obtained mutants were fully sequenced (Eurofins Genomic, Les Ulis, France), amplified, and purified (#740490, Macherey-Nagel, Hœrdt, France). Plasmid concentrations were measured using a Nanodrop (Thermo Fisher Scientific, Illkirch-Graffenstaden, France).

### 4.2. Cell Culture and Transfection

HEK293 cells were purchased from ATCC and cultivated in DMEM medium (#C31966-021) supplemented with 10% fetal calf serum (#10270-106) (Thermo Fisher Scientific, Illkirch-Graffenstaden, France). Cells were maintained at 37 °C, 5% CO_2_. For western blot and cell surface analysis, cells were seeded in 6-well plates and transfected with 1.5 µg CFTR plasmids using Lipofectamine 3000 (#L3000-015, Thermo Fisher Scientific, Illkirch-Graffenstaden, France). For halide sensitive YFP assay, cells were transfected using Lipofectamine 3000 (#L3000-015, Thermo Fisher Scientific, Illkirch-Graffenstaden, France) with a total of 1.5 µg of plasmid, either 1.3 µg of YFP plasmid and 0.2 µg of CFTR-WT/CFTR-L188W plasmids or 0.75 µg of YFP plasmid and 0.75 µg of other mutant CFTR plasmids.

### 4.3. Western Blot Analysis

The day after transfection, cells were treated with 3 µM of VX-809 (#S1565, Selleckchem, Planegg, Germany) for 24 h before being lysed in RIPA buffer containing protease inhibitors (#04693124001, Roche Life Science, Basel, Switzerland) and protein concentration assessed using RcDc assay (#5000113, BioRad, Marnes-la-Coquette, France). Western blot analysis was performed using 60 µg of protein from each sample separated on 7% SDS-PAGE gels. After transfer onto nitrocellulose membranes, CFTR was probed using antibody 660 (Cystic Fibrosis Foundation, Chapel Hill, NC, USA) and alpha-tubulin probed with antibody DM1A (#sc32293, SantaCruz, Dallas, TX, USA). Quantification of band intensities was performed using ImageJ software (version 1.53c, NIH, Bethesda, MD, USA).

### 4.4. Halide Sensitive Functional Assay

CFTR activity was measured in transiently transfected HEK293 cells using the halide-sensitive yellow fluorescent protein YFP-H148Q/I152L [24]. The day after transfection, cells were transferred to poly-L-lysine- (#P4707, Sigma-Aldrich, Saint-Quentin-Fallavier, France) coated 96 well black/clear bottom microplates (#655-09, Greiner-BioOne, Les Ulis, France). After 24 h, plates were washed with PBS, and each well was incubated for 30 min with 100 µL of PBS containing cpt-AMPc (#c3912, 100 µM) and IBMX (#I5879, 100 µM) (all from Sigma-Aldrich, Saint-Quentin-Fallavier, France) with and without 10 µM VX-770 (#s1144, Selleckchem, Planegg, Germany) alone or in combination with 20 µM apigenin (#10798, Sigma-Aldrich, Saint-Quentin-Fallavier, France). Plates were then transferred to a ClarioStar plate-reader (BMG Labtech, Ortenberg, Germany) equipped with an injector, which enabled continuous recording of fluorescence (YFP filters) during injection. After 3 s, 200 µL of PBS-NaI (PBS solution where NaCl is replaced with NaI) was injected. Cell fluorescence recordings were normalized to the initial average value measured, and signal decay was fitted to an exponential function to derive the maximal slope. Maximal slopes were converted to rates of change in intracellular I-concentration (in mM/s).

### 4.5. Cell Surface Quantification

CFTR cell surface was measured using hIBIT complementation (N2420, Promega, Charbonnières-les-Bains, France). This assay is based on the reconstitution of the nanoluciferase activity, which is split in two parts: an eleven amino acid peptide (smBIT) and a large subunit (lgBIT). Transfected HEK-293 cells were plated in white 96-well plates, and 24 h after transfection, cells were washed and incubated with a recombinant lgBIT and furimazin substrate to measure cell surface CFTR. Cells were then permeabilized using Triton x100 (#T9284, Sigma-Aldrich, Saint-Quentin-Fallavier, France), inducing the diffusion of the LgBIT inside the cells and enabling quantification of the total amount of CFTR. Quantification was performed by quantifying the Surface-CFTR/Total-CFTR ratio. Detection of the produced luminescent was performed on a Mitras plate reader (Berthold, Thoiry, France).

### 4.6. Statistical Analysis

Quantitative variables were described as mean (± SEM). Comparisons to WT conditions and between treated and untreated conditions were made with Mann–Whitney test for *p* evaluation.

### 4.7. Molecular Dynamics Simulations/3D Structure Analysis

The 3D structures were visualized with the UCSF Chimera package [39].

For MD simulations, the 3D structure of human CFTR (pdb:6MSM) was embedded in a 1-palmytoyl-2-oleoyl-phosphatidylcholine (POPC) bilayer and solvated in a 150 mM NaCl solution. The CHARMM36 force field [40] was used for the protein, lipids, and ions, and the TIP3P model for water. Minimization, equilibration, and production steps were performed on the occigen/cines supercomputer using Gromacs 2019.1 [41]. The standard CHARMM-GUI inputs [42] were used for the minimization and equilibration of the systems. During these steps, harmonic restraints applied to the protein heavy atoms and the lipid heads and were gradually released during 1.2 ns. The production dynamics were then performed in the NPT ensemble without any restraints. A Nose–Hoover thermostat [43] and Parrinello–Rahman barostat [44] were used to keep the temperature and the pressure constant at 310 K and 1 bar. Periodic boundary conditions were used, and the particle mesh ewald algorithm was applied to treat long range electrostatic interactions [45]. A switching function was applied between 10 and 12 Å for the non-bonded interactions. LINCS [46] was applied to constrain the bond lengths involving hydrogen atoms. The integration timestep was set to 2 fs, and the overall length of the trajectory was 700 ns.

## Data Availability

Data available on request.
